# Clinically Relevant Influenza Virus Evolution Reconstituted in a Human Lung Airway-on-a-Chip

**DOI:** 10.1128/Spectrum.00257-21

**Published:** 2021-09-15

**Authors:** Longlong Si, Haiqing Bai, Crystal Yuri Oh, Lei Jin, Rachelle Prantil-Baun, Donald E. Ingber

**Affiliations:** a Wyss Institute for Biologically Inspired Engineering at Harvard University, Boston, Massachusetts, USA; b Harvard John A. Paulson School of Engineering and Applied Sciences, Harvard University, Cambridge, Massachusetts, USA; c Vascular Biology Program and Department of Surgery, Boston Children’s Hospital, Harvard Medical School, Boston, Massachusetts, USA; Johns Hopkins Hospital

**Keywords:** influenza virus, viral evolution, lung airway-on-a-chip, preclinical model, drug resistance, gene reassortment

## Abstract

Human-to-human transmission of viruses, such as influenza viruses and coronaviruses, can promote virus evolution and the emergence of new strains with increased potential for creating pandemics. Clinical studies analyzing how a particular type of virus progressively evolves new traits, such as resistance to antiviral therapies, as a result of passing between different human hosts are difficult to carry out because of the complexity, scale, and cost of the challenge. Here, we demonstrate that spontaneous evolution of influenza A virus through both mutation and gene reassortment can be reconstituted *in vitro* by sequentially passaging infected mucus droplets between multiple human lung airway-on-a-chip microfluidic culture devices (airway chips). Modeling human-to-human transmission of influenza virus infection on chips in the continued presence of the antiviral drugs amantadine or oseltamivir led to the spontaneous emergence of clinically prevalent resistance mutations, and strains that were resistant to both drugs were identified when they were administered in combination. In contrast, we found that nafamostat, an inhibitor targeting host serine proteases, did not induce viral resistance. This human preclinical model may be useful for studying viral evolution *in vitro* and identifying potential influenza virus variants before they appear in human populations, thereby enabling preemptive design of new and more effective vaccines and therapeutics.

**IMPORTANCE** The rapid evolution of viruses, such as influenza viruses and severe acute respiratory syndrome coronavirus 2 (SARS-CoV-2), is challenging the use and development of antivirals and vaccines. Studies of within-host viral evolution can contribute to our understanding of the evolutionary and epidemiological factors that shape viral global evolution as well as development of better antivirals and vaccines. However, little is known about how viral evolution of resistance to antivirals occurs clinically due to the lack of preclinical models that can faithfully model influenza infection in humans. Our study shows that influenza viral evolution through mutation or gene reassortment can be recapitulated in a human lung airway-on-a-chip (airway chip) microfluidic culture device that can faithfully recapitulate the influenza infection *in vitro.* This approach is useful for studying within-host viral evolution, evaluating viral drug resistance, and identifying potential influenza virus variants before they appear in human populations, thereby enabling the preemptive design of new and more effective vaccines and therapeutics.

## INTRODUCTION

The emergence of more infectious viral mutants represents a formidable challenge to the battle against viral pandemics, as has been observed during the current COVID-19 crisis where new severe acute respiratory syndrome coronavirus 2 (SARS-CoV-2) variants continue to be identified that exhibit enhanced replication and transmission as well as potential reduced responsiveness to existing vaccines (1). Similar rapid viral evolution during human-to-human transmission occurs in many other airborne pathogens, including influenza viruses. Virus evolution often results from accumulation of gene mutations due to misincorporation of nucleotides by the viral RNA-dependent RNA polymerase (RdRP) during genome replication that result in amino acid substitutions, a process known as antigenic drift (2). However, influenza viruses can also undergo dramatic genome changes leading to antigenic drift when two or more different strains combine to form a new subtype through gene reassortment (3). These two mechanisms enable viruses to escape host immune responses, causing major concerns to public health. Development of more effective approaches to confront viral pandemics will, therefore, require better prediction of evolution of virus resistance to therapy and more rapid development of novel drugs and vaccines, both of which are currently limited by the lack of clinically relevant preclinical models (2). Here, we explored whether human organ-on-a-chip (organ chip) microfluidic culture technology (4–7) can be used to tackle this challenge. Our approach is based on recent demonstration that influenza A virus strain-dependent virulence and multifaceted host responses to infection can be modeled in a human lung airway chip (8), which also had been previously shown to replicate multiple other disease states as well as drug responses and toxicities *in vitro* (5, 7).

## RESULTS

**FIG 1:**
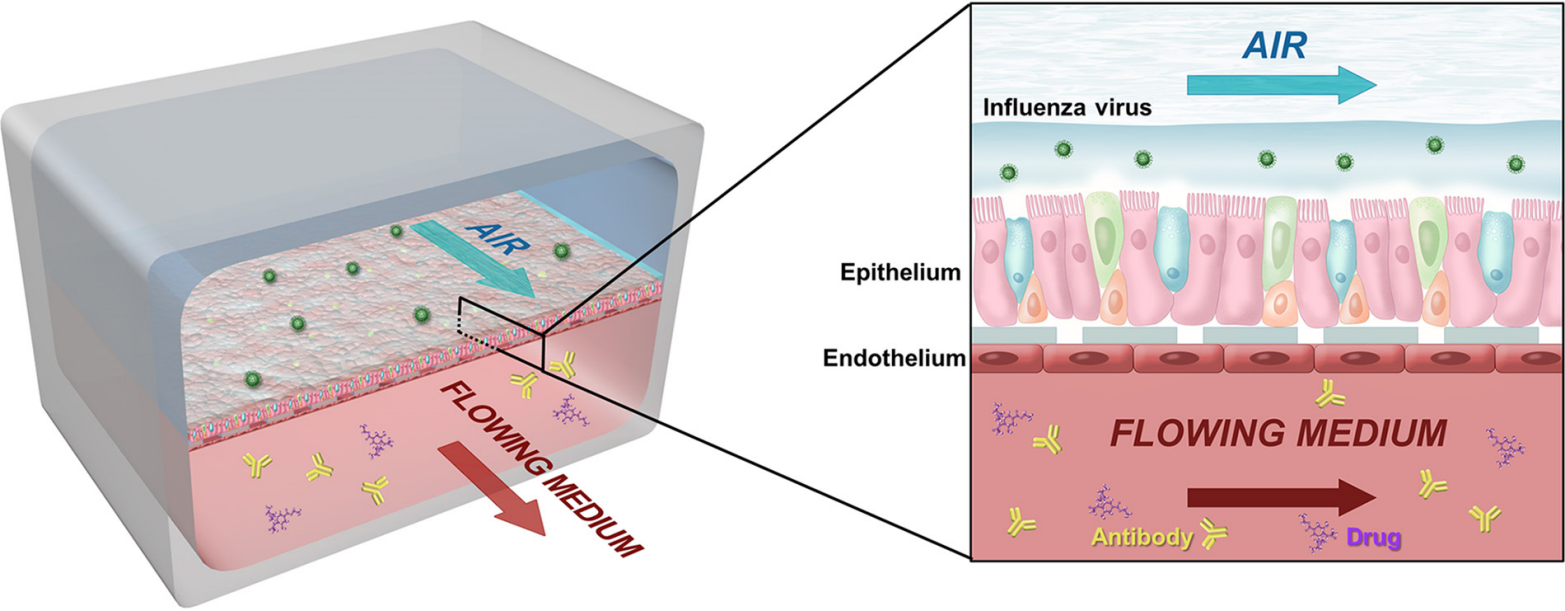
Schematic diagram of human airway chip. Human airway chip is a two-channel microfluidic cell culture device composed of an air channel and a blood channel. The air channel is lined by highly differentiated human primary airway cells cultured under an air-liquid interface; the blood channel is lined by human pulmonary endothelial cells. Influenza virus is introduced to the air channel to mimic the airborne route of transmission. Drugs and antibodies can be perfused via the blood channel to mimic drug treatment.

**FIG 2:**
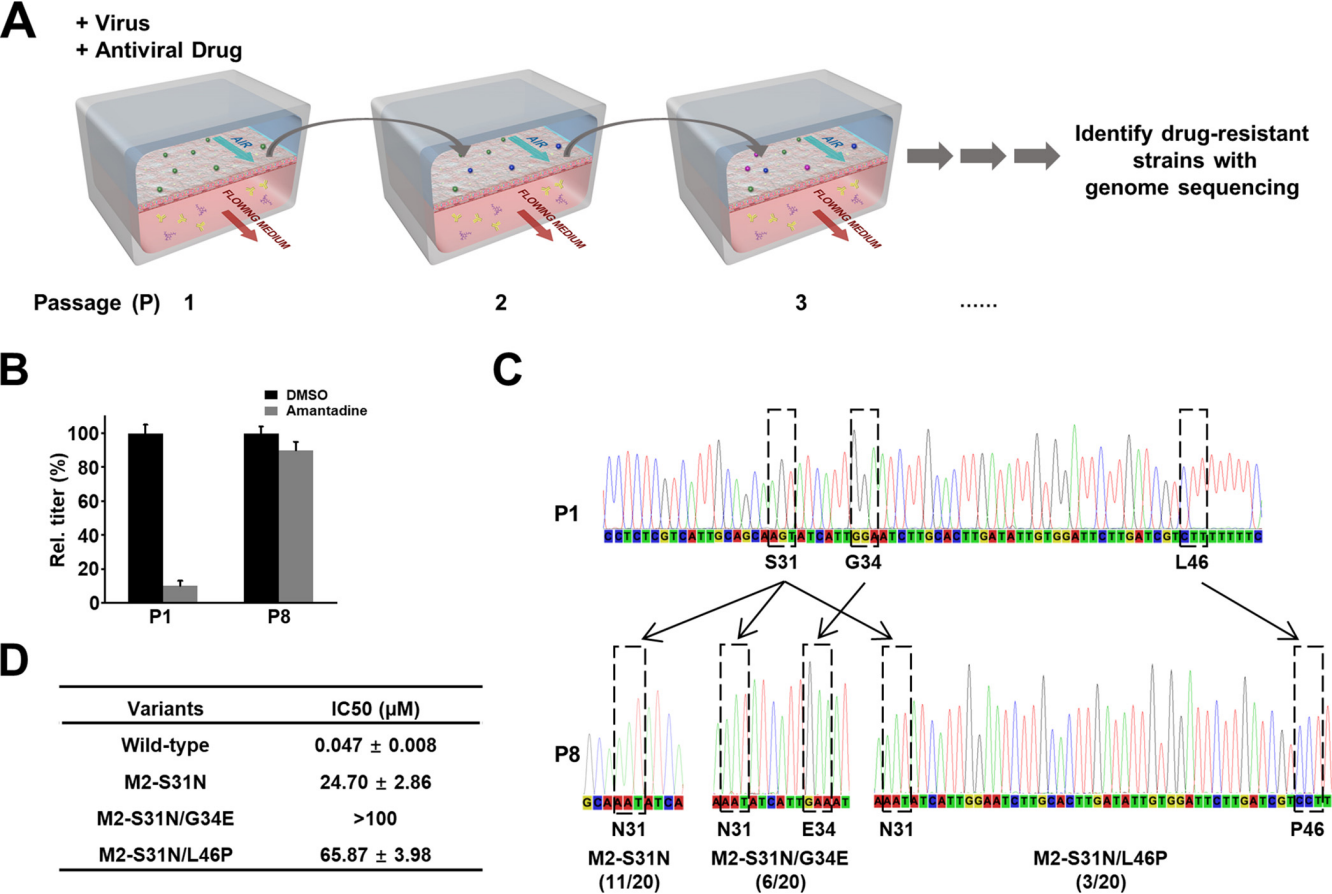
Modeling influenza virus evolution through mutation on-chip. (A) Schematic diagram of method used to generate and identify drug-resistant viruses by human chip-to-chip transmission under drug pressure. (B) Plaques and graph showing plaque titers of progeny virus at 1st and 8th passage (P) in control (dimethyl sulfoxide [DMSO]) versus amantadine-treated chips. (C) Sequencing graphs showing three mutants (M2-S31N, M2-S31N/G34E, and M2-S31N/L46P) detected in the amantadine-resistant virus pool (the proportion of each mutant is noted in parentheses). (D) IC_50_ values of amantadine against parental strain and the three mutants.

The human lung airway chip is an optically clear silicone rubber device that contains two parallel microchannels separated by an extracellular matrix (ECM)-coated porous membrane lined by primary human lung airway epithelial cells (HLAECs) cultured under an air-liquid interface (ALI) on one side within the “airway” channel, with human pulmonary microvascular endothelial cells (HPMVECs) grown on the other in the presence of continuous medium flow to mimic vascular perfusion with or without human immune cells within the “blood” channel (Fig. 1). To explore whether the human airway chip viral infection model can be used to model virus evolution in human populations, we passaged influenza A/WSN/33 (H1N1) virus from chip to chip in the presence of clinically used antiviral drugs (either amantadine or oseltamivir) to mimic human-to-human transmission of influenza virus in patients treated with these drugs (Fig. 2A). Viral genome sequencing was carried out every other passage to monitor the spontaneous emergence of viral mutations.

When we treated each sequentially infected human airway chip with amantadine at a dose (1 μM) that inhibited replication of this influenza strain by ∼90% under initial conditions, the drugs became progressively less effective with increased passaging. Amantadine was only able to produce ∼10% inhibition after eight rounds of human chip-to-chip passaging (Fig. 2B), indicating the emergence of a pool of amantadine-resistant viruses. Genome sequencing of these drug-resistant viruses led to the identification of three different mutated virus strains with mutation sites located within the viral M2 protein (Fig. 2C), which is the known target of amantadine (9). Among them, a single mutation at S31N conferred amantadine resistance with the half maximal inhibitory concentration (IC_50_) increasing over 500-fold (from 47 nM to 24.7 μM) (Fig. 2D). Importantly, this is the same mutation that has been frequently detected in patients with clinically documented amantadine-resistant influenza viruses (10). In addition, we identified two double mutants that contained the S31N mutation, as well as either G34E or L46P substitutions (Fig. 2C). The M2 G34E mutation has been previously observed in human transmissions of influenza virus infections associated with amantadine resistance (11). The M2 L46P mutation has not been reported in human clinical isolates, but after publication of an initial preprint describing our findings (12), an *in vitro* serial viral passage study reported the same mutation in response to treatment with isoxazole-conjugated amantadine (13). These mutations conferred even greater resistance to amantadine, with the IC_50_ increasing over 1,000-fold (from 47 nM to >100 or 65 μM, respectively) (Fig. 2D), indicating that extended exposure to amantadine has the potential to induce emergence of virus strains that are even more highly resistant to therapy.

**FIG 3:**
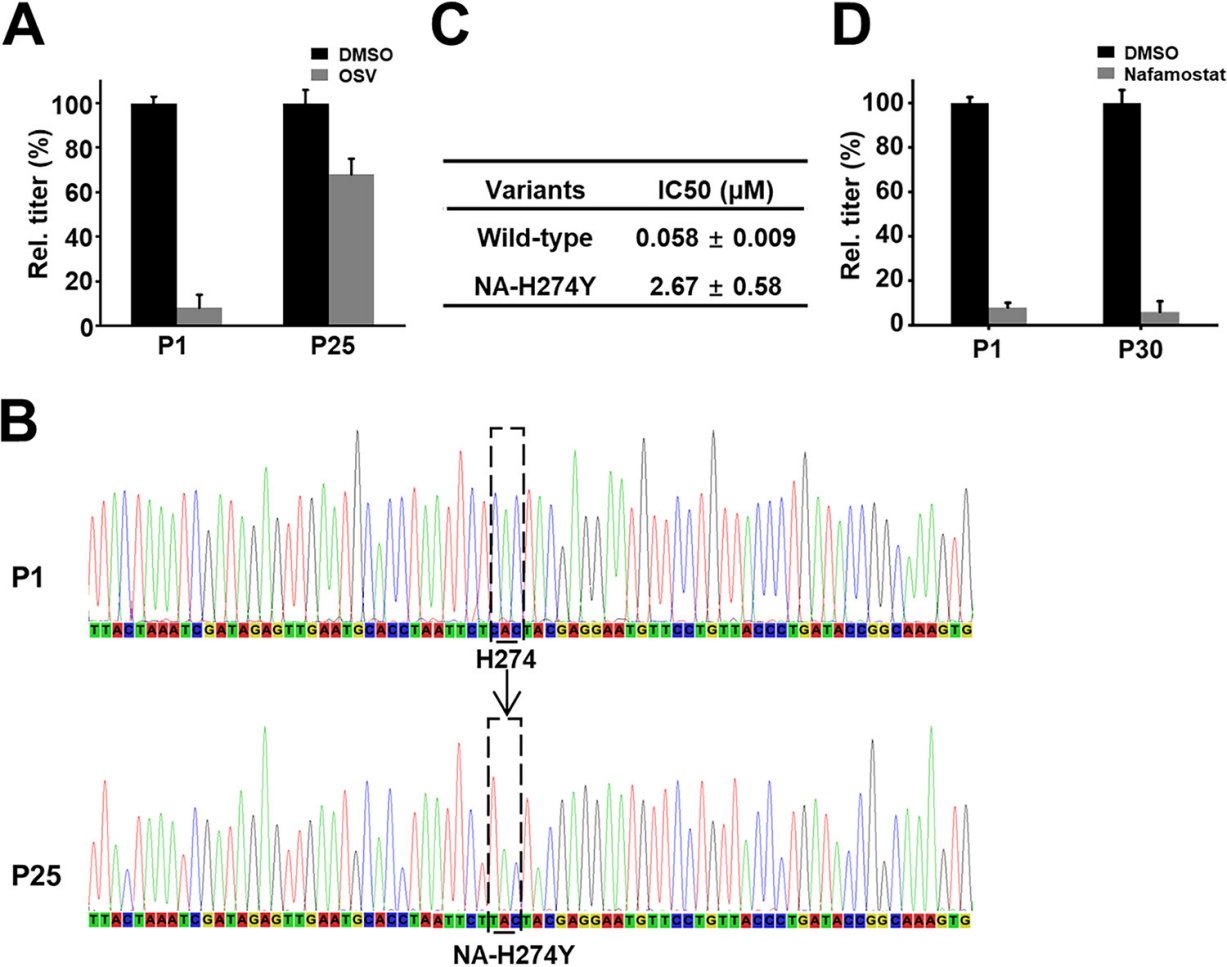
Comparison of the ability of oseltamivir (OSV) and nafamostat to induce viral resistance on-chip. (A) Graph showing plaque titers of progeny virus at 1st and 25th passage in control (DMSO) versus oseltamivir-treated chips. (B) Sequencing graphs showing one mutant (NA-H274Y) detected in the OSV-resistant virus pool. (C) IC_50_ values of OSV against parental strain and mutant. (D) Graph showing plaque titers of progeny virus at 1st and 30th passage in control (DMSO) and nafamostat-treated chips and the lack of development of drug resistance.

Next, we used the same approach to explore whether influenza H1N1 virus can develop resistance to the widely used anti-influenza drug oseltamivir (Tamiflu) *in vitro*. These studies revealed that oseltamivir’s ability to inhibit H1N1 infection in human airway chips also decreased significantly (from ∼90% to ∼30%) over time (Fig. 3A); however, this was a much slower process, as 25 human chip-to-chip passages were required for resistance to develop (Fig. 3B). Sequencing of the oseltamivir-resistant virus pool revealed one strain with an H274Y mutation site located within the influenza viral neuraminidase (NA) protein that is the known target of oseltamivir (Fig. 3B) (14). This mutation conferred oseltamivir resistance, with the IC_50_ increasing from 58 nM to 2.67 μM (Fig. 3C), and again, the same mutation has been previously detected in clinical cases (14).

Furthermore, the host serine protease inhibitor nafamostat, which has been shown to have potential as an anti-influenza therapeutic (8), did not induce viral resistance even when virus infection was transmitted through 30 chip-to-chip passages in the presence of this drug (Fig. 3D). Importantly, this result is in dire contrast to the results that we obtained with the viral protein-targeting antiviral drugs, amantadine and oseltamivir, which are currently used clinically and induce emergence of drug-resistant virus strains on-chip (Fig. 2 and 3) as they do in patients (10, 14).

**FIG 4:**
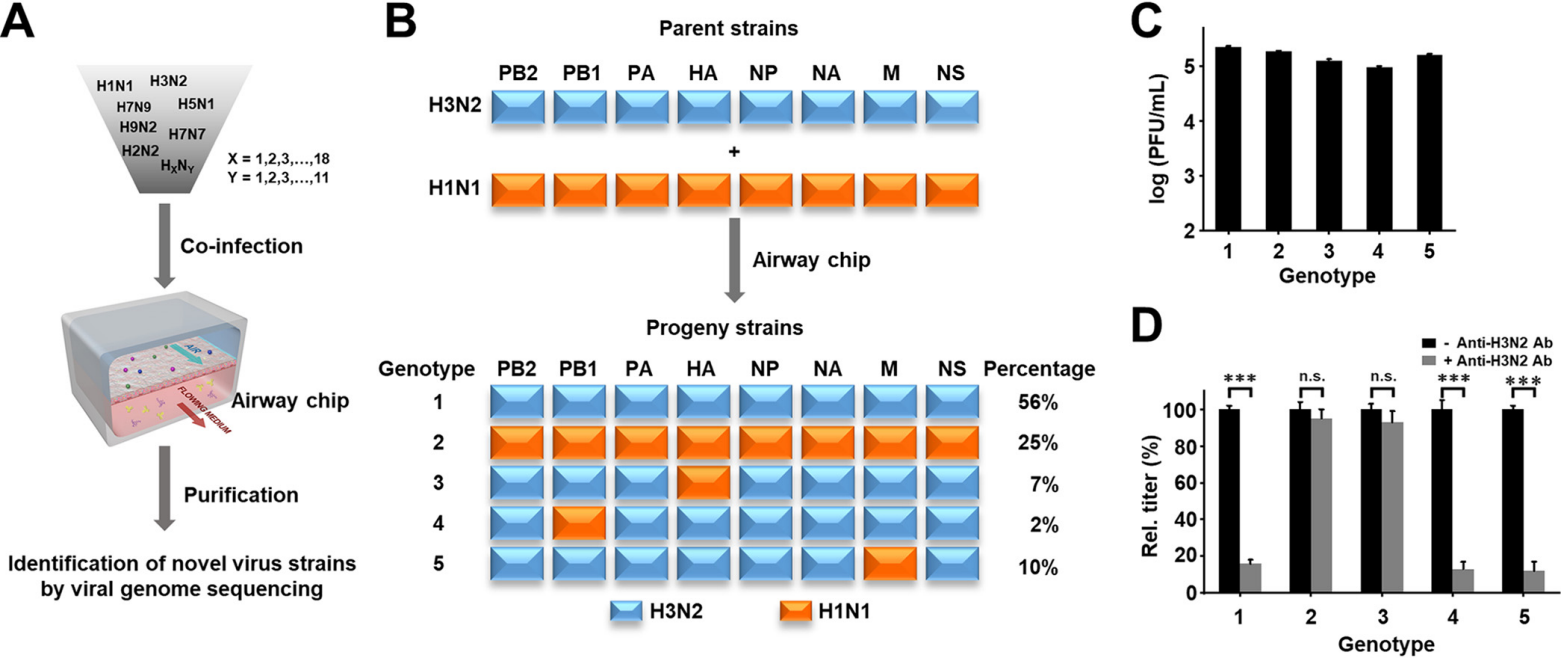
Modeling influenza virus evolution through gene reassortment on-chip. (A) Schematic diagram of the method for generation and identification of reassortants in airway chips coinfected with different strains. (B) Five genotypes with different percentages of incidence revealed from sequencing analysis of 100 progeny viruses isolated from airway chips coinfected by pandemic influenza A/Netherlands/602/2009 (H1N1) and seasonal influenza A/Panama/2007/99 (H3N2) viruses (blue boxes, segments derived from H3N2; orange boxes, segments from H1N1). (C) Replication titers of different genotypes of reassortants and their parental strains in airway chips 48 h postinfection (MOI = 0.1). (D) The neutralization activity of anti-H3N2 HA antibody (10 μg/ml) against different genotypes of reassortants and their parental strains. ***, *P* < 0.001; n.s., not significant.

To explore whether the airway chip could also support influenza virus antigenic shift through gene reassortment as occurs when different virus strains coinfect the same host (15), airway chips were coinfected with seasonal influenza A/Panama/2007/99 (H3N2) and pandemic influenza A/Netherlands/602/2009 (H1N1) viruses, cultured for 2 days, and their progeny viruses were then isolated and sequenced (Fig. 4A). Genotype analysis of 100 progeny viruses revealed that the H3N2 and H1N1 parental strains were predominant; however, 19 virus reassortants emerged that represented three distinct genotypes (3, 4, and 5) (Fig. 4B). These included seven H1N2 reassortants containing the hemagglutinin (HA) gene segment from H1N1 with other gene segments from H3N2 in genotype 3, two new H3N2 reassortants containing the polymerase basic protein-1 (PB1) gene segment from H1N1 with others from H3N2 in genotype 4, and 10 new H3N2 reassortants containing the matrix protein (M) gene segment from H1N1 with others from H3N2 in genotype 5 (Fig. 4B). These reassortants did not exhibit higher replication competence than their parental strains (Fig. 4C), but the emergence of antigenically drifted H1N2 reassortants led to complete resistance to binding of an H3N2-specific anti-HA antibody (Fig. 4D), indicating the potential risk of pandemic development if antigen-mismatched vaccination occurred. Importantly, the H1N2 reassortant predicted on-chip also has been detected in patients during influenza season in which H1N1 and H3N2 cocirculated (15).

**FIG 5:**
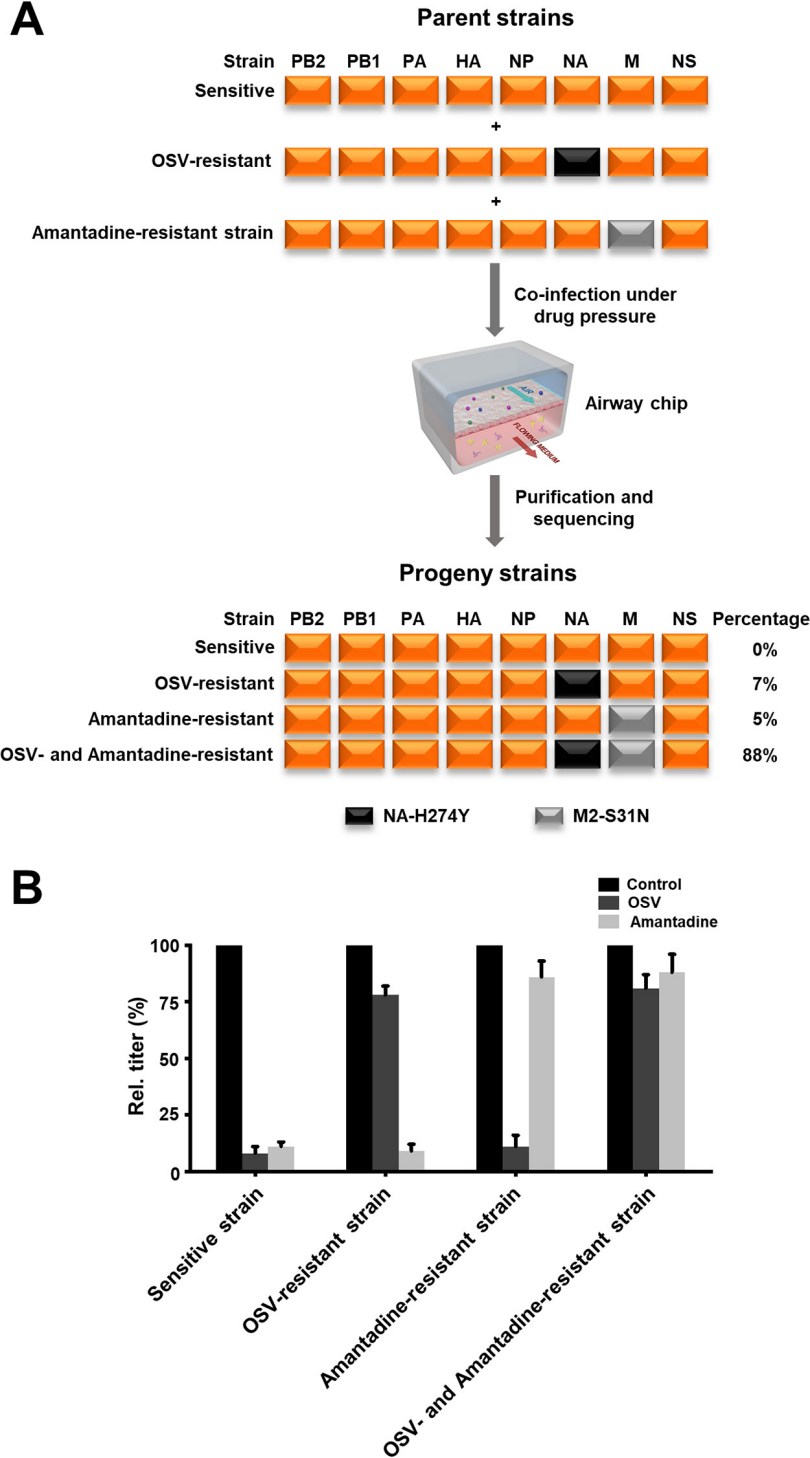
Generation of an influenza virus stain that is double resistant to both oseltamivir and amantadine through reassortment in the airway chip. (A) When drug-sensitive, OSV-resistant, and amantadine-resistant WSN (H1N1) influenza viruses (MOI = 1.0) were used to coinfect the same human airway chip in the presence of OSV and amantadine (1 μM), sequencing analysis of 100 progeny viruses isolated from the coinfected chips revealed that all progeny exhibited drug resistance and 3 genotypes were identified (%, relative incidence). Orange boxes indicate segments from the drug-sensitive virus strain; black boxes indicate segments from the OSV-resistant strain; gray boxes indicate segments from the amantadine-resistant virus strain. (B) Virus titer detection showing activity of OSV and amantadine (1 μM) against parental virus strain and the progeny reassortants.

A cocktail of antiviral drugs can be used in clinic to circumvent rapid viral mutation due to single mutation. To examine if airway chips enable discovery of viral mutations to drug combinations, we combined the drug-sensitive WSN influenza strain with oseltamivir-resistant and amantadine-resistant WSN viruses and infected the Airway Chips with all three virus strains in the presence of both drugs (Fig. 5A). Sequencing analysis revealed a high frequency (88%) of double-resistant viruses as a result of gene reassortment (Fig. 5A). The NA-H274Y and M2-S31N double mutant variant that emerged was able to completely evade treatment with a combination of both amantadine and oseltamivir (Fig. 5B).

## DISCUSSION

Taken together, these results clearly demonstrate that virus evolution through mutation or gene reassortment, as well as during treatment with antiviral drug combination therapy, can be studied *in vitro* using human airway chips. Importantly, the airway chip can faithfully reproduce evolution of drug-resistant influenza virus strains that previously emerged in human populations and are clinically documented (9, 10, 14). Our results showing that evolution of drug-resistant variants occurs more easily in the presence of amantadine than oseltamivir are also consistent with clinical observations, and this is a major reason why amantadine is no longer used as a first-line drug in the clinic. The demonstration that viruses that are doubly resistant to both drugs can emerge when both drugs are administered simultaneously is concerning and should be taken into account in future clinical investigations. At the same time, our finding that nafamostat does not induce emergence of resistant viruses in this study is intriguing, and this is likely due to the fact that it targets the host cell response rather than molecular targets on the rapidly replicating virus. Nafamostat is a serine protease inhibitor used as an anticoagulant drug in the clinic that was recently shown to also inhibit influenza infection by blocking the cleavage of influenza hemagglutinin protein (HA0) into the subunits HA1 and HA2 by serine proteases (e.g., TMPRSS11D and TMPRSS2), which is essential for viral entry (8, 16). Successful evasion from host-targeted therapies requires extensive mutational changes in the pathogen because host factors are evolutionarily conserved, and thus the virus has a high genetic barrier to overcome (17, 18). Taken together, these results suggest that this experimental human preclinical platform, therefore, potentially could be used to predict viruses that are resistant to current antiviral drugs or vaccines, which might emerge in the future. Moreover, by combining the influenza evolution prediction in airway chips with existing influenza surveillance approaches (15, 19–21), it might be possible to establish public databases of influenza evolution in humans, which could lead to a better selection of candidate viruses for vaccines and early detection of drug-resistant viruses (22).

Various computational models for predicting the influenza virus evolution have been developed that are based on the relationship between genetic mutations and antigenic characteristics of circulating viruses via global surveillance; however, their accuracy is suboptimal, and it varies from year to year (23). Antigenic characterization of circulating viruses using ferret antisera is still the mainstay in vaccine strain selection because ferrets have respiratory tracts that are more “human-like” than other animal models, but ferret immunity is significantly different from that of humans. For example, human and ferret antibodies elicited via the same process target different epitopes of the influenza HA (24). Thus, the airway chip model may serve as a human alternative to the ferret model to improve the prediction of influenza viral evolution in addition to being able to study host immune response to viral infection as recently demonstrated (8).

Viral evolution experiments can be conducted in eggs or cell culture (25, 26); however, the host innate immune response plays an essential role in constraining virus evolution by decreasing the cells’ ability to support virus replication and by reducing the virus diversity on which selection can operate (3). For this reason, in a human challenge study in which volunteers were inoculated with viral stocks that had been grown in eggs and cell culture, many of the variants that had emerged through mutation during passage in culture were purged from the viral population during or shortly after inoculation due to host selection pressure (27, 28). Because the airway chip enables more faithful mimicry of host innate immune response and host selection pressure compared to traditional cell line models of influenza (8), it provides an alternative approach to study viral evolution *in vitro*, which might circumvent this limitation and thereby better enable identification of clinically relevant variants and novel strains.

It is important to note that in contrast to static air-liquid interface cultures, we were able to administer oseltamivir and amantadine via dynamic perfusion through the endothelium-lined vascular channel of the device, which allows us to mimic their delivery via systemic administration through the vasculature in patients. This dynamic mode of drug administration on-chip has been shown to replicate the therapeutic time window of oseltamivir observed in patients *in vivo* (8). We and others also have shown that fluid shear stress in the microfluidic human airway chip leads to physiologically relevant mucociliary velocities and coordinated mucociliary movement, rather than random motions, which may play a role in viral entry and clearance (5, 29). In addition, infection of these chips with the same strain of influenza H1N1 virus as used in the present study has been shown to elicit a host cytokine response that closely replicates host responses observed in human patients (8), which also may affect viral replication dynamics. Furthermore, as different drug pharmacokinetic profiles can be replicated in microfluidic organ chips (30), use of this model can enable one to explore the effects of different drug exposure profiles and administration routes on development of drug resistance, which is not possible with static models.

One limitation of the current study is the lack of neutrophils and other adaptive immune cells during our evaluation of viral evolution; however, they can be easily incorporated via the blood channel, as we have demonstrated previously (8). More importantly, by being able to carry out these studies in the absence of immune cells, we show that their presence is not required for the evolution of drug-resistant viruses that have been previously observed in human patients. However, future studies could be carried out to determine how incorporating additional selective pressures (e.g., pulmonary macrophages, neutrophils, fibroblasts, etc.) might impact viral fitness and, consequently, more complex patterns of viral evolution. Finally, while our study focuses on the evolution of influenza viruses, similar approaches can be adopted by labs with biosafety level 3 (BSL3) capabilities to investigate SARS-CoV-2 evolution and could greatly enrich our arsenal against this and future pandemic viruses.

## MATERIALS AND METHODS

### Human airway chip culture.

Microfluidic organ chip devices from Emulate Inc. (Boston, MA) were used for constitution of the airway chips as recently described (8). Briefly, chips were activated by ER1 solution (Emulate Inc.), washed in ER2 buffer (Emulate Inc.), and placed under a UV lamp (NailStar; NS-01-US) for 20 min. The channels were then washed sequentially with ER2 buffer and Dulbecco’s phosphate-buffered saline (DPBS). The porous membranes were coated on both sides with collagen type IV from human placenta (0.5 mg/ml; Sigma-Aldrich) at room temperature overnight. The solution was then aspirated before seeding cells. Primary HLAECs (Lonza, USA) obtained from healthy donors 448571, 446317, 623950, 485960, and 672447) and primary HPMVECs (Cell Biologics, USA) were cultured as described previously (8). The human airway chips were created as described previously (8).

### Influenza virus infection in the human airway chip.

The procedure has been described previously (8). Briefly, human airway chips were infected with influenza viruses by flowing 30 μl of phosphate-buffered saline (PBS) containing the indicated multiplicity of infection (MOI) of viral particles into the apical channel, incubating for 2 h at 37°C, and then removing the medium to reestablish an ALI. To measure virus propagation, the apical channel was incubated with 50 μl of PBS for 1 h at 37°C, and then the apical fluid was collected from the apical channels to quantify viral load using the plaque formation assay.

### Identification of drug-resistant virus strains.

Airway chips were infected with amantadine- and oseltamivir-sensitive influenza A/WSN/33 virus (MOI = 0.01) and treated with 1 μM amantadine (Sigma-Aldrich), 1 μM oseltamivir acid, 10 μM nafamostat (Abcam), or left untreated for 48 h. Amantadine or oseltamivir acid was perfused through the vascular channel under flow (60 μl/h). Nafamostat was delivered into the apical airway channel. The progeny viruses were collected by incubating the airway channel with 50 μl PBS for 1 h at 37°C and collecting the fluid. Progeny virus yields were quantified using the plaque formation assay after each passage (virus yield of untreated airway chips was set as 100%), and then the same number of progeny viral particles (MOI = 0.01) were used for each round of subsequent infection in new airway chips. This procedure was repeated up to 30 times. When the progeny virus pool became resistant to drug treatment, the viruses were isolated through plaque purification and gene sequenced to identify gene mutations according to the standard viral genome confirmation approach under molecular genetics provided by Genewiz.

### Generation and identification of influenza reassortants.

Airway chips were coinfected with pandemic influenza A/Netherlands/602/2009 (H1N1) virus (MOI = 2) and seasonal influenza A/Panama/2007/99 (H3N2) virus (MOI = 2) for 48 h, and then the progeny virus pool was collected by washing the airway channel with 50 μl PBS. The progeny viruses were isolated through plaque purification and gene sequenced to investigate any reassortment that occurred in their genomes. To evaluate the effects of reassortment on viral replication competence, parental virus strains and isolated reassortants were used to infect airway chips (MOI = 0.1), and their replication titers were quantified using the plaque formation assay 48 h later. To evaluate the effects of reassortment on the efficacy of anti-HA (H3N2) antibody (Sino Biological), parental virus strains and isolated reassortants were used to infect the human airway chip (MOI = 0.1) in the presence or absence of the antibody (10 μg/ml), and their replication titers were measured using the plaque formation assay.

To study reassortment between wild-type and drug-resistant viruses in the airway chip, wild-type, oseltamivir-resistant, and amantadine-resistant influenza/A/WSN/33 (H1N1) virus strains obtained from the experiment shown in Fig. 2 were used to infect airway chips (MOI = 1) in the presence of oseltamivir and amantadine (1 μM). Two days later, their progeny viruses were isolated by plaque purification and gene sequenced according to the standard viral genome confirmation approach under molecular genetics provided by Genewiz.

### Statistical analysis.

Tests for statistically significant differences between groups were performed using a two-tailed unpaired Student’s *t* test. Differences were considered significant when the *P* value was less than 0.05 (*, *P* < 0.05; **, *P* < 0.01; ***, *P* < 0.001; n.s., not significant). All results are expressed as means ± standard deviation (SD); error bars indicated *n* > 3.
